# Effect of nano-silicon on the regulation of ascorbate-glutathione contents, antioxidant defense system and growth of copper stressed wheat (*Triticum aestivum* L.) seedlings

**DOI:** 10.3389/fpls.2022.986991

**Published:** 2022-10-13

**Authors:** Muhammad Riaz, Shaopeng Zhao, Muhammad Kamran, Naveed Ur Rehman, Freddy Mora-Poblete, Carlos Maldonado, Muhammad Hamzah Saleem, Aasma Parveen, Abdullah Ahmed Al-Ghamdi, Fahad M. Al-Hemaid, Shafaqat Ali, Mohamed S. Elshikh

**Affiliations:** ^1^ College of Resources and Environment, Zhongkai University of Agriculture and Engineering, Guangzhou, China; ^2^ College of Natural Resources and Environment, South China Agricultural University, Guangzhou, China; ^3^ School of Agriculture, Food and Wine, The University of Adelaide, Adelaide, SA, Australia; ^4^ Provincial Key Laboratory of Plant Molecular Breeding, South China Agricultural University, Guangzhou, China; ^5^ Institute of Biological Sciences, University of Talca, Talca, Chile; ^6^ Centro de Genómica y Bioinformática, Facultad de Ciencias, Universidad Mayor, Santiago, Chile; ^7^ College of Plant Science and Technology, Huazhong Agricultural University, Wuhan, China; ^8^ Department of Soil Science, Faculty of Agriculture and Environmental Sciences, The Islamia University of Bahawalpur, Bahawalpur, Pakistan; ^9^ Department of Botany and Microbiology, College of Science, King Saud University, Riyadh, Saudi Arabia; ^10^ Department of Environmental Sciences and Engineering, Government College University, Faisalabad, Pakistan; ^11^ Department of Biological Sciences and Technology, China Medical University, Taichung, Taiwan

**Keywords:** wheat, silicon, oxidative stress, AsA-GSH, copper toxicity

## Abstract

Copper (Cu^2+^) toxicity can inhibit plant growth and development. It has been shown that silicon (Si) can relieve Cu^2+^ stress. However, it is unclear how Si-nanoparticles (SiNPs) relieve Cu^2+^ stress in wheat seedlings. Therefore, the current study was conducted by setting up four treatments: CK, SiNP: (2.5 mM), Cu^2+^: (500 µM), and SiNP+Cu^2+^: (2.5 mM SiNP+500 µM Cu^2+^) to explore whether SiNPs can alleviate Cu^2+^ toxicity in wheat seedlings. The results showed that Cu^2+^ stress hampered root and shoot growth and accumulated high Cu^2+^ concentrations in roots (45.35 mg/kg) and shoots (25.70 mg/kg) of wheat as compared to control treatment. Moreover, Cu^2+^ treatment inhibited photosynthetic traits and chlorophyll contents as well as disturbed the antioxidant defense system by accumulating malondialdehyde (MDA) and hydrogen peroxidase (H_2_O_2_) contents. However, SiNPs treatment increased root length and shoot height by 15.1% and 22%, respectively, under Cu^2+^ toxicity. Moreover, SiNPs application decreased MDA and H_2_O_2_ contents by 31.25% and 19.25%, respectively. SiNPs increased non-enzymatic compounds such as ascorbic acid-glutathione (AsA-GSH) and enhanced superoxide dismutase (SOD), peroxidase (POD), catalase (CAT), and ascorbic peroxidase (APX) activities by 77.5%, 141.7%, 68%, and 80%, respectively. Furthermore, SiNPs decreased Cu^2+^ concentrations in shoots by 26.2%, as compared to Cu^2+^ treatment alone. The results concluded that SiNPs could alleviate Cu^2+^ stress in wheat seedlings. The present investigation may help to increase wheat production in Cu^2+^ contaminated soils.

## Introduction

In agroecosystems, heavy metals are continuously accumulated in the soil and water. The phenomenon can be attributed to several anthropogenic activities. This includes modern farming practices, such as extensively exploiting the resources of the earth and industrialization (Zhang et al., 2019a). Copper (Cu^2+^), as a micronutrient, is essential for plant growth and development (Droppa and Horváth, 1990) and has a wide range of applications in metal manufacturing and pesticide production, which has attracted much attention in recent years (Saleem et al., 2020). Cu^2+^ is essential for metabolism, enzymes, and proteins, and maintains structural and catalytic activity during normal plant growth (Emamverdian et al., 2015). In addition, the accumulation of Cu^2+^ in plants has the potential to pose a serious threat to human health through the food chain. Cu^2+^ is ranked fourth among the most widely distributed inorganic pollutants according to a report published by the Chinese Ministry of Environment Protection. The assessment of agricultural soil for Cu^2+^ contamination at 1731 sites nationwide found that 21.02% of the sampling sites surpassed the screening value (50 mg/kg) (Li et al., 2020). In soil, Cu^2+^ concentrations are in the range of 14–109 mg/kg while 5–30 mg/kg Cu^2+^ of plant DW is regarded as ideal for crop productivity (Kabata-Pendias, 2000). However, Cu^2+^ toxicity can negatively affect the cellular and physiological developmental processes of plant metabolism (Peñarrubia et al., 2015). Cu^2+^ toxicity not only leads to the accumulation of reactive oxygen species (ROS) but also hinders plant growth (Hall, 2002; Adrees et al., 2015a). Cu^2+^ stress induces oxidative stress (oxidative metabolism) and destroys macromolecules and even causes cell death in severe cases (Noctor et al., 2018). Sulfhydryl groups in membrane proteins can be affected by excessive Cu^2+^ stress, resulting in the deformations of cell membranes (Emamverdian et al., 2015). The only way for organisms to survive deleterious oxidative stress is through evolving an effective and efficient system of antioxidants, e.g., superoxide dismutase (SOD), catalase (CAT), peroxidases (POD), proline, carotenoids, glutathione (GSH) and ascorbic acid (AsA) (Das and Roychoudhury, 2014; Hasan et al., 2018; Ali et al., 2022). For optimal growth, it is essential to coordinate antioxidants (enzymatic and non-enzymatic) to maintain ROS levels at stable levels, especially under metal contaminations (Noctor et al., 2018). Consequently, the defense system is enhanced in the plant body with the help of exogenous chemicals (Gill and Tuteja, 2010) which is an effective way of fighting unnecessary ROS levels caused by metal contamination (Yi et al., 2018; Zhang et al., 2019b). Therefore, it is necessary to implement remedial measures and/or strategies to reduce Cu^2+^ accumulation and uptake in plants, thus ensuring crop productivity and reducing possible health risks caused by Cu^2+^ accumulation.

Silicon (Si) accounts for 28% of the earth’s crust and is found as silica, silicic acid, and silicates (Singer and Munns, 1987). It has been reported that exogenous Si treatment ameliorates heavy metal stress by inducing antioxidative responses in the plant body (Emamverdian et al., 2018; Bhat et al., 2019). Silicon improves the defense system by reducing ROS and oxidative stress (Adrees et al., 2015b; Anwaar et al., 2015; Sabir et al., 2022). Silicon addition increased plant growth, development, and membrane stability under heavy metal stress (Rahman et al., 2017). As a regulator, Si reduces metal uptake and transport by plants and improves physiological adaptability and resource usage under heavy metal stress (Ahmad et al., 2021). Nanotechnology has become a valuable tool for agriculture (Jiang et al., 2021). There is no doubt that it has found widespread application in agriculture due to sustainable agriculture and precise farming (Prasad et al., 2017). Several studies have demonstrated the potential benefits of silicon nanoparticles (SiNPs) in the past few years on plant growth by mitigating abiotic and biotic stresses occurring in plants due to adverse environmental factors (Roychoudhury, 2020). It has been shown that SiNPs can be more effective in reducing heavy metal toxicity in plants in comparison with normal Si (Bhat et al., 2019).

Throughout the world, wheat (*Triticum aestivum* L.) is the second most cultivated crop and is found in almost all countries. Cu^2+^ is important in wheat production. However, Cu^2+^ toxicity can inhibit wheat productivity and Cu^2+^ can be readily transferred to the grain. In contrast, little information is available on the role of SiNPs in plant tolerance to Cu^2+^. The mechanism for reducing Cu^2+^ toxicity in wheat seedlings through SiNPs application remains unknown. Silicon can play a significant role in soil remediation, hence a deeper knowledge of SiNPs and Cu^2+^ absorption and combined action is needed. Therefore, the objectives of this study were to investigate how SiNPs can play a beneficial role in reducing Cu^2+^ toxicity by enzymatic (SOD, POD, CAT) and non-enzymatic (AsA-GSH) antioxidant defense systems, and to study how SiNPs can reduce Cu^2+^ concentrations in plant tissues, which may help to reduce Cu^2+^ toxicity in wheat seedlings by improving growth parameters. The results of this study may help to increase the yield of wheat in Cu^2+^ contaminated soil.

## Methods and materials

### Plant material and experimental procedures

Wheat (*Triticum aestivum*) “cultivars-97003” was used as test material. The wheat seedlings were grown in hydroponics at South China Agriculture University, China. The seeds were washed with 10% H_2_O_2_ for 20 min and then rinsed with distilled water three times. Seeds were spread on wet clothes for germination. After one week of emergence, seedlings were transferred to pots filled with ¼ nutrient strength for a week and then subsequently 1/3 and finally to full strength nutrient solution (mM); “2.0 (NH_4_)_2_SO_4_, 12.0 NaNO_3_, 1.0 NaH_2_PO_4_·2H_2_O, 3.0 K_2_SO_4_·5H_2_O, 3.0 MgSO_4_·2H_2_O, 4.0 CaCl_2_, (μM), 9.15 MnCl_2_·4H_2_O, 0.32 CuSO_4_·5H_2_O, 0.77 ZnSO_4_·7H_2_O, 0.02 (NH_4_)_6_Mo_7_O_24_·4H_2_O, 46.26 H_3_BO_3_, and 20.0 FeSO_4_·7H_2_O-EDTA”. The 12 seedlings were planted in a 2 litter pot, totaling 16 pots. The following treatments were set up in the experiment, (i) CK: normal nutrient solution; (ii) SiNP: 2.5 mM; (iii) Cu^2+^: 500 µM (iv) SiNP+Cu^2+^: 2.5 mM+500 µM. Copper was applied as CuSO_4_.5H_2_O. The experiment had 4 treatments and each treatment was replicated four times in a completely randomized design. The experimental treatments were selected based on our trial experiments where we used different concentrations of Cu^2+^ and SiNP and then finally selected suitable treatments (500 µM Cu^2+^ and 2.5 mM SiNP) for the current experiment. During the present study, we used silicon nanoparticles (SiNPs), which have been characterized in our previous study (Riaz et al., 2022). The morphology of SiNP was almost spherical and its size was between 12-17 nm. Every three days, the nutrient solution was changed throughout the experiment, pH was maintained at 6.5 daily and samples were taken out after two weeks. In order to clean the roots of the seedling, distilled water was used to wash the surface after taking out the seedlings. The water in the roots of the seedlings was gently blotted with absorbent paper, and the junction of the rhizomes and stems was used as the node to measure the plant height and root length of each wheat plant and recorded in cm. Wheat seedlings were separated from the junction of rhizomes and stems, into the aerial parts and underground parts, and fresh weight (FW) was measured. The samples were stored at -80°C and the remaining samples were oven-dried at 68° C until constant weight.

### Photosynthetic parameters involved in gas exchange

A portable infrared gas analyzer was used to record the parameters related to gas exchange “Li-6400, Li-Cor, Inc., Lincoln, NE, USA”. We selected only the leaves that were fully expanded. We measured net photosynthetic rates (Pn), transpiration rates (Tr), internal CO_2_ concentrations (Ci), and stomatal conductance (gs). During measurements, 360 µmol mol^-1^ was CO_2_ concentrations and 1000 µmol m^-2^ was photosynthetic photon flux density s^-1^ at 25 °C.

### Chlorophyll contents in wheat plants

Chlorophyll contents in leaves were determined by the method of Lichtenthaler (1987). As part of the investigation, we determined the contents of chlorophyll in fully expanded leaf tissues. The 200 mg wheat leaf samples after rinsing with deionized water were chopped and treated with 95% alcohol. The mixture was centrifuged at 3000 rpm for 5 min and then chlorophyll contents were measured by a spectrophotometer at 665, 649, and 470 nm wavelengths.

### Contents of MDA and H_2_O_2_

To determine the MDA content in the samples, the method of De Vos et al. (1991) was used. The 0.5 g of root sample was measured and after homogenizing the samples, 8 mL of 50 mmol/L PBS (PH=7.8) was added and centrifuged at 10000 r/min for 10 min. Next, 2.5 ml of TBA solution was added and plugged with a stopper, placed in boiling water for 20 min at 98 °C, and centrifuged at 4000 r/min for 10 min after cooling. The absorbance values were measured at 532, 600, and 450 nm wavelengths.

For the measurement of H_2_O_2_ content, the method reported by Velikova et al. (2000) was used. Briefly, liquid nitrogen was used to crush fresh root samples (0.2 g) and homogenized with a trichloroacetic acid solution of 0.1% (1 mL) and after that homogenate was centrifuged at 12,000 r/min for 20 min (4°C) and the supernatant was collected. In the reaction mixture, 1 mL of BPS (pH 6.8, 100 mM) was added, as well as 0.5 mL of supernatant containing 1 mL of KI (1M). The contents of H_2_O_2_ were measured employing a spectrophotometer (UV-VIS 2550, Shimadzu, Japan) at 390 nm wavelength.

### The determination of SOD, POD, CAT, and APX activities

The superoxide dismutase (SOD) activity was determined according to the method of Beauchamp and Fridovich (1971). The 0.5 g root sample was homogenized with PBS (PH 7.8) and centrifuged at 10,000 r/min for 10 min at 4°C. Subsequently, supernatant and reagents were mixed and shaken at 4°C and placed under fluorescent light for 25 min. The light-proof test tube was used as a blank, and the absorbance value was measured at 560 nm by a spectrophotometer.

The determination of POD activity refers to the method of Cai et al. (2008). The 0.5 g of root samples were taken into a centrifuge tube and then 10 mL of PBS (PH 7.8) was added and homogenized by a cooled mortar and pestle. The homogenate was centrifuged at 8000 r/min for 10 min at 4°C, and then the supernatant was taken and mixed with the reaction solution. The absorbance was measured at 470 nm by spectrophotometer, and the reading was taken at 30s, 90s, and 150s. CAT activity was measured as described by Aebi (1983).

To determine the activity of APX, the method of Nakano and Asada (1981) was used. For this, 0.5 g of root sample was homogenized in a liquid N_2_ and taken into a 15 mL centrifuge tube. Then, 10 mL of pre-cooled extract was added with the reagent and centrifuged at 15000 r/min, 4°C for 15 min, and the supernatant was used for the determination of APX activity at 290 nm by spectrophotometer.

### Measurement of non-enzymatic antioxidants

Ascorbate (AsA) and contents of dehydroascorbate (DHA) were measured as reported by Gillespie et al. (2007) with some modifications. Briefly, the roots were homogenized by adding 1.6 mL of 6% trichloroacetic acid (TCA). Afterward, the homogenate was centrifuged at 13,000 rpm for 5 min, 4 ◦C. The absorbance was measured at a wavelength of 525 nm. A standard curve was used to estimate the total ascorbate content of the solution, and DHA concentrations were determined based on deducting the reduced ascorbate value from the total ascorbate value. The contents of glutathione (GSH) and oxidized glutathione (GSSG) were assessed, and detection kits were used for this purpose (A061-1) (Nanjing Jiancheng, China).

### Measurement of Cu^2+^ and Si concentrations in roots and shoots

Oven-dried root and shoot samples of wheat were ground to a fine powder and then 0.3 g of corresponding samples were put in a mixture of HNO_3_:HClO_4_ (6 mL) (5:1 *v*/*v*) overnight and digested on a hotplate at 120 °C until 1 mL liquid was left (Keller et al., 2015). The liquid was cooled, and the final volume was made up to 50 mL by adding deionized water, and Cu^2+^ and Si concentrations were measured by Atomic Absorption Spectrophotometer (AAS) “AA6300C, Shimadzu, Japan”. Translocation factor of Cu^2+^ was calculated as “TF = Metal concentration in shoot (mg/kg DW)/Metal concentrations in roots (mg/kg DW)”.

### Statistical analysis

Data were analyzed by one-way ANOVA, and the significant differences among different treatments were compared by LSD test (P<0.05), using the protocols given by Steel et al. (1997). Values are mean ± standard deviation (SD) of at least four replicates and graphed using Origin 8.0 software.

## Results

### Effect of SiNPs on growth traits of wheat seedlings under Cu^2+^ stress

**Figure 1:**
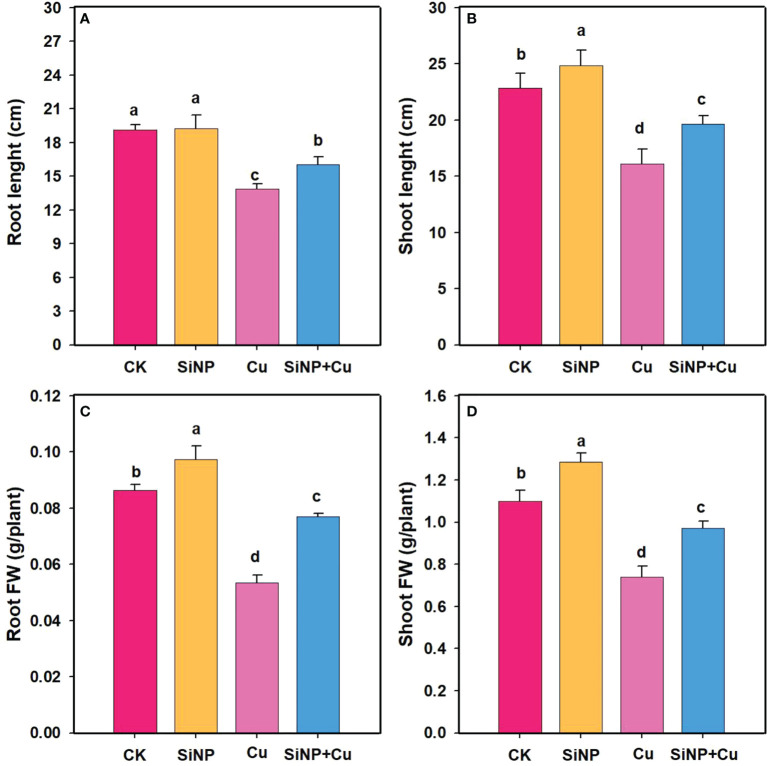
The growth characters of wheat seedlings under Cu^2+^ stress. Note “Experimental treatments contained: CK (Control); SiNP (2.5 mM SiNPs); Cu^2+^ (500 µM Cu^2+^ alone), SiNP+Cu^2+^ (2.5 mM SiNPs+500 µM Cu^2+^). The different letters (a, b, c, d) above bars describe the differences in different treatments by LSD test at (P ≤ 0.05)”. **(A)** root length, **(B)** shoot length, **(C)** root FW, **(D)** shoot FW.

The growth characteristic of wheat seedlings under Cu^2+^ stress is shown in **Figure 1**. As compared to the control treatment, Cu^2+^ stress alone significantly reduced root length and shoot height by 27.48% and 29.46%, respectively (**Figures 1A, B**). On the other hand, under SiNPs+Cu^2+^ treatment, shoot height increased by 22.04%, and root length increased by 15.61%. Cu^2+^ treatment had a significant negative effect on the fresh biomass of wheat seedlings and decreased the fresh weight of roots and shoots by 38% and 32.7% respectively. However, SiNPs+Cu^2+^ treatment reduced the inhibition effect of Cu^2+^ on the growth parameters of wheat seedlings and increased root and shoot fresh weight (**Figures 1C, D**).

### Chlorophyll contents under Cu^2+^ stress

**Figure 2:**
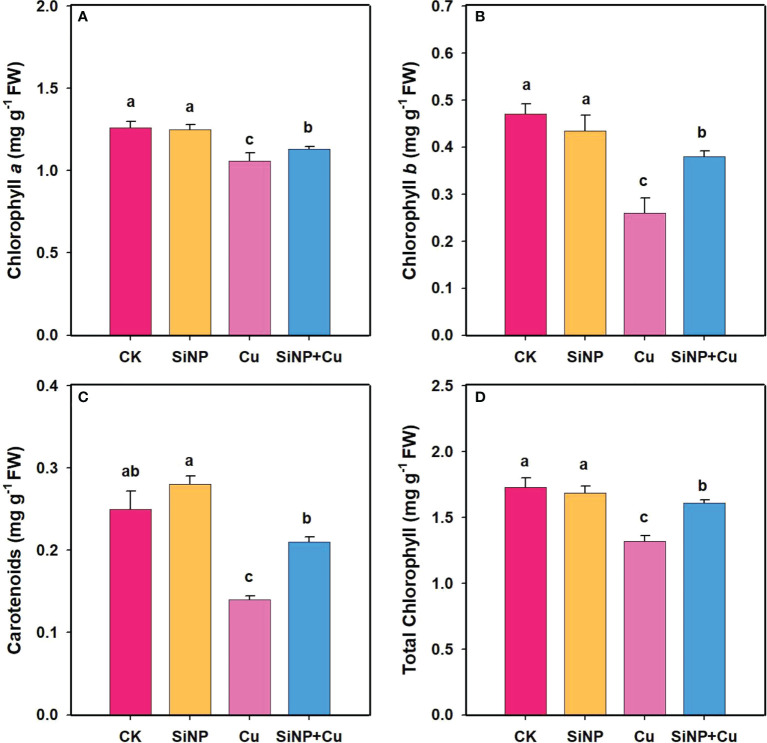
Chlorophyll contents of wheat seedlings under Cu^2+^ stress. Note **“**Experimental treatments contained: CK (control); SiNP (2.5 mM SiNPs); Cu (500 µM Cu^2+^ alone), SiNP+Cu (2.5 mM SiNPs+500 µM Cu^2+^). The different letters (a, b, c, d) describe the differences in different treatments by LSD test at (P ≤ 0.05)**”**. **(A)** chlorophyll a, **(B)** chlorophyll b, **(C)** carotenoids, **(D)** total chlorophyll.

Chlorophyll contents in leaves of wheat seedlings under each treatment are shown in **Figure 2**. Cu^2+^ treatment decreased chlorophyll *a* by 15.87%, chlorophyll *b* by 44.68% (**Figures 2A, B**), carotenoids by 44%, and total chlorophyll by 23.69% compared to the control treatment (**Figures 2C, D**). Compared to Cu^2+^ treatment, SiNP+Cu^2+^ significantly increased chlorophyll *a*, *b*, carotenoids, and total chlorophyll contents by 6.6%, 46.15%, 50%, and 21.9%, respectively.

### Effects of SiNPs on gas exchange traits of wheat leaves under Cu^2+^ stress

**Figure 3:**
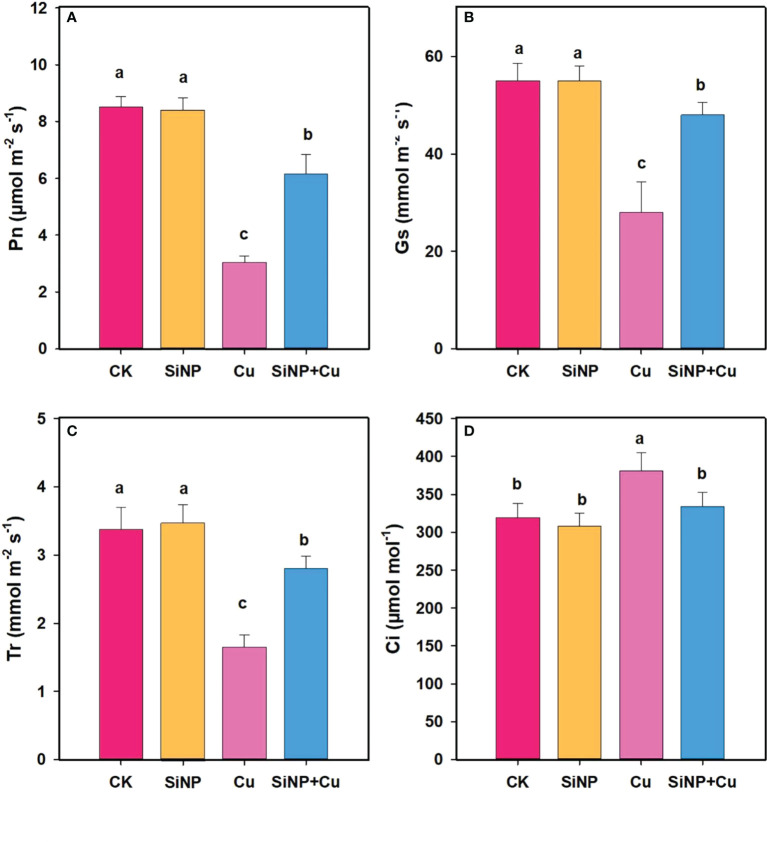
Gas exchange traits under Cu^2+^ stress. Note **“**Experimental treatments contained: CK (control); SiNP (2.5 mM SiNPs); Cu (500 µM Cu^2+^ alone), SiNP+Cu (2.5 mM SiNPs+500 µM Cu^2+^). The different letters (a, b, c, d) describe the differences in different treatments by LSD test at (P ≤ 0.05)**”**. **(A)** Pn, **(B)** Gs, **(C)** Tr, **(D)** Ci.

Compared with the control, Cu^2+^ treatment alone significantly reduced gas exchange traits of wheat leaves (**Figure 3**) while SiNP+Cu^2+^ reversed the effect of Cu^2+^ on inhibition of gas exchange traits of wheat leaves. SiNPs+Cu^2+^ treatment increased the Pn (102.63%), gs (71.42%) (**Figures 3A, B**), and Tr (69.69%) while decreasing Ci (12.33%) of wheat leaves, respectively, compared with Cu^2+^ alone (**Figures 3C, D**).

### Effects of SiNPs on MDA and H_2_O_2_contents in roots of wheat under Cu^2+^ stress

**Figure 4:**
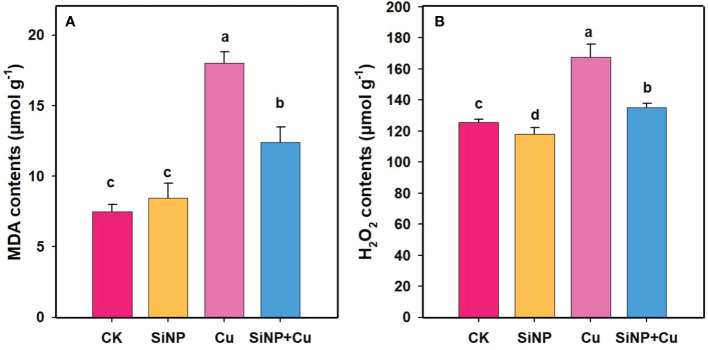
MDA and H_2_O_2_ contents of wheat roots under Cu^2+^ stress. Note **“**Experimental treatments contained: CK (control); SiNP (2.5 mM SiNPs); Cu (500 µM Cu^2+^ alone), SiNP+Cu (2.5 mM SiNPs+500 µM Cu^2+^). The different letters (a, b, c, d) describe the differences in different treatments by LSD test at (P ≤ 0.05)**”**. **(A)** MDA contents, **(B)** H2O2 contents.

Malondialdehyde (MDA) and H_2_O_2_ contents in wheat root seedlings were determined, which are presented in **Figure 4**. The results showed that MDA and H_2_O_2_ content were increased by 140.80% and 33.20%, respectively, under Cu^2+^ treatment alone in comparison to control treatment. However, SiNP+Cu^2+^ treatment significantly reduced MDA and H_2_O_2_ content by 31.25% and 19.25% in the roots of wheat seedlings, respectively, compared with Cu^2+^ treatment alone, indicating reduced damage to membranes and reduced oxidative stress (**Figures 4A, B**).

### Effects of SiNPs on enzymatic activities on roots of wheat under Cu^2+^ stress

**Figure 5:**
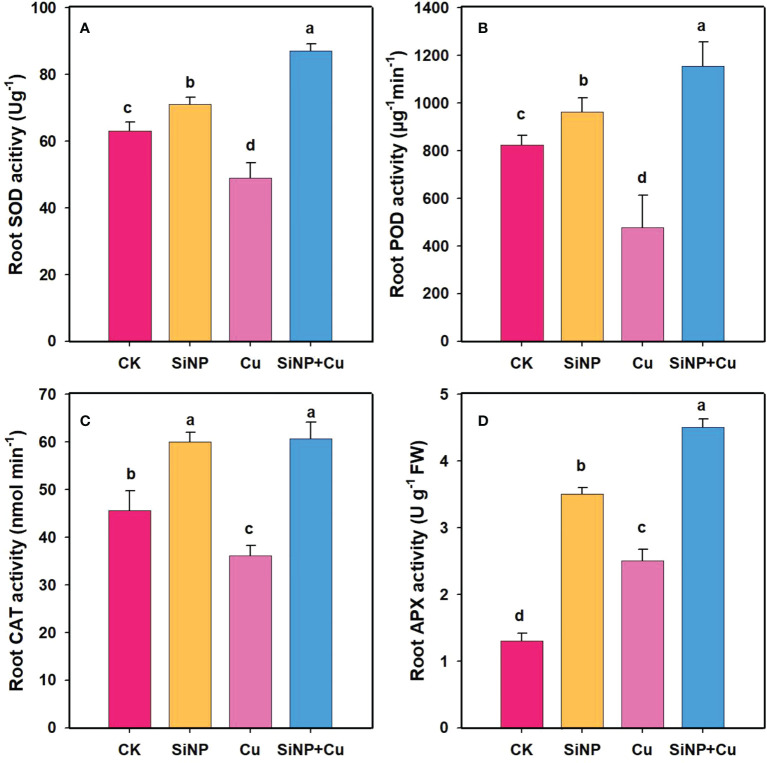
Enzymatic activities under Cu^2+^ stress. Note **“**Experimental treatments contained: CK (control); SiNP (2.5 mM SiNPs); Cu (500 µM Cu^2+^ alone), SiNP+Cu (2.5 mM SiNPs+500 µM Cu^2+^). The different letters (a, b, c, d) describe the differences in different treatments by LSD test at (P ≤ 0.05)**”**. **(A)** SOD, **(B)** POD, **(C)** CAT, **(D)** APX.

Cu^2+^ treatment decreased the SOD activity in roots by 22.2% compared to CK (**Figure 5**). However, SiNP+Cu^2+^ treatment increased SOD enzyme activity by 77.5% related to Cu^2+^ treatment alone (**Figure 5A**). It can be seen from **Figure 5** that the POD enzyme activity of wheat roots was inhibited under Cu^2+^ treatment by 42% while SiNPs+Cu^2+^ treatment increased POD activity by 141.71% as compared to Cu^2+^ treatment (**Figure 5B**). Moreover, SiNP+Cu^2+^ treatment increased the activity of CAT (**Figure 5C**) and APX enzymes by 68% and 80% respectively, as compared to Cu^2+^ treatment alone (**Figure 5D**).

### Effects of SiNPs on AsA-GSH contents under Cu^2+^ stress

**Figure 6:**
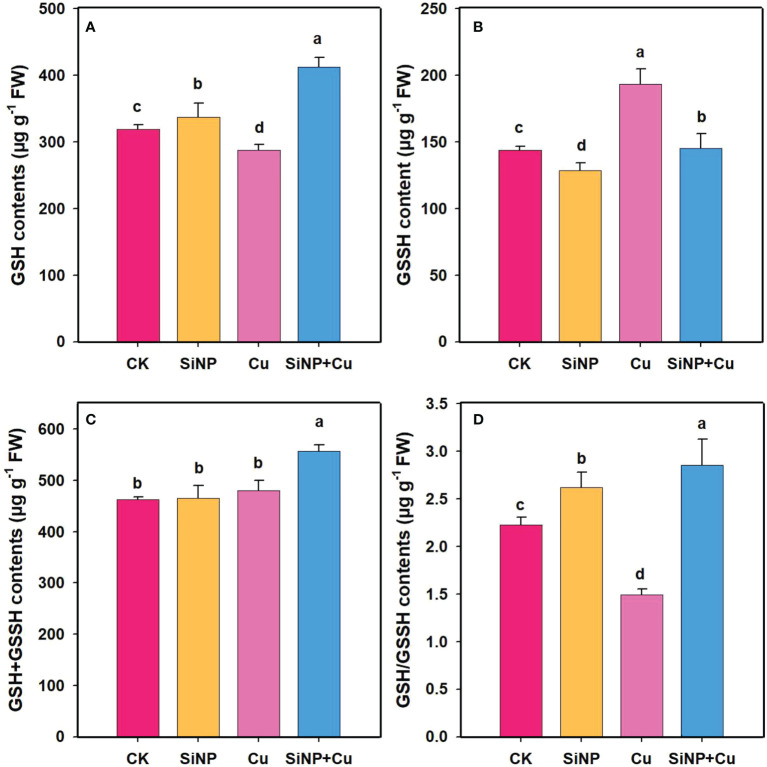
GSH contents under Cu^2+^ toxicity. Note **“**Experimental treatments contained: CK (control); SiNP (2.5 mM SiNPs); Cu (500 µM Cu^2+^ alone), SiNP+Cu (2.5 mM SiNPs+500 µM Cu^2+^). The different letters (a, b, c, d) describe the differences in different treatments by LSD test at (P ≤ 0.05)**”**. **(A)** GSH, **(B)** GSSH, **(C)** GSH+GSSH, **(D)** GSH/GSSH.

**Figure 7:**
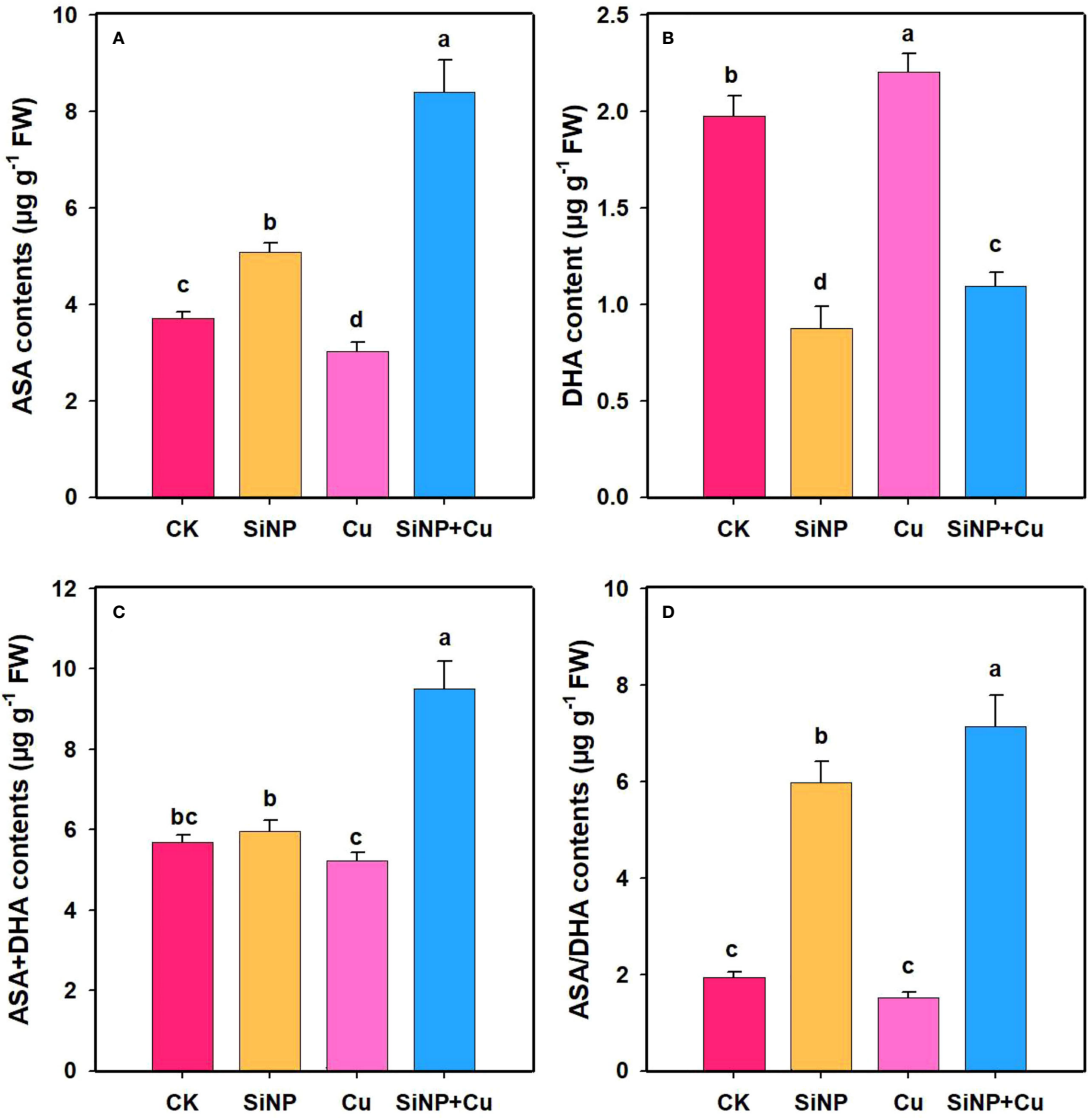
ASA contents under Cu^2+^ toxicity. Note **“**Experimental treatments contained: CK (control); SiNP (2.5 mM SiNPs); Cu (500 µM Cu^2+^ alone), SiNP+Cu (2.5 mM SiNPs+500 µM Cu^2+^). The different letters (a, b, c, d) describe the differences in different treatments by LSD test at (P ≤ 0.05)**”**. **(A)** AsA, **(B)** DHA, **(C)** AsA+DHA, **(D)** AsA/DHA.

To explore the role of SiNPs on AsA-GSH contents under Cu^2+^ stress, the contents of non-enzymatic substances in wheat roots were determined (**Figures 7A–D**). Compared with control, Cu^2+^ stress decreased GSH and AsA contents by 9.87% (**Figure 6A**) and 18.42% (**Figure 7A**) but increased GSSG and DHA contents by 34.6% and 11.53% in wheat roots. SiNP+Cu^2+^ treatment significantly increased GSH and AsA contents by 43.39% and 178% and decreased GSSG (**Figure 6B**) and DHA contents by 24.83% and 50.34%, respectively as compared to Cu^2+^ treatment alone (**Figure 7B**).

### Effect of SiNPs on Cu^2+^ and Si accumulation in various wheat tissues

**Figure 8:**
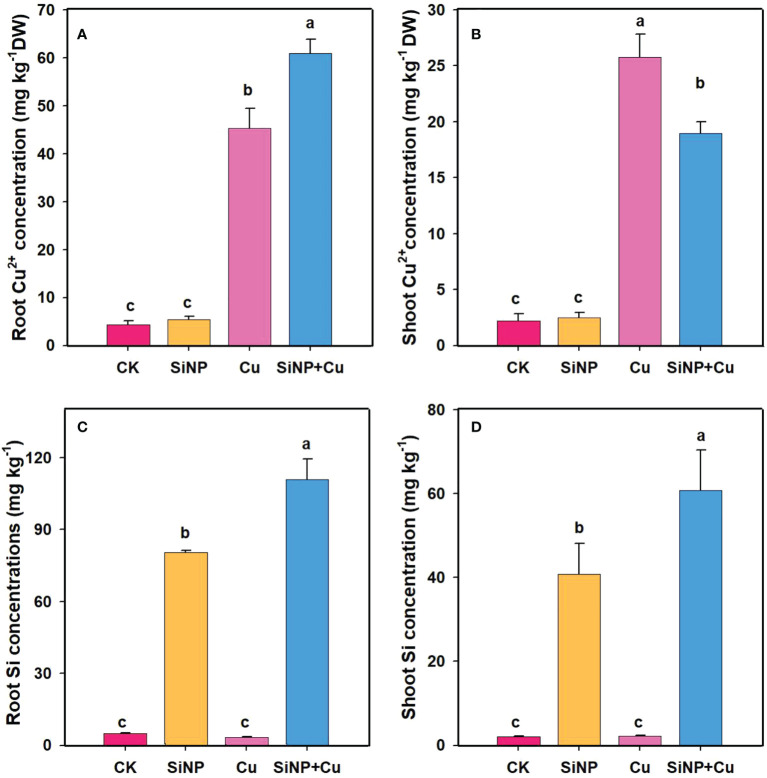
Cu^2+^ and Si contents of wheat seedlings under Cu^2+^ stress. Note **“**Experimental treatments contained: CK (control); SiNP (2.5 mM SiNPs); Cu (500 µM Cu^2+^ alone), SiNP+Cu (2.5 mM SiNPs+500 µM Cu^2+^). The different letters (a, b, c, d) describe the differences in different treatments by LSD test at (P ≤ 0.05)**”**. **(A)** root Cu2+, **(B)** shoot Cu2+, **(C)** Root Si, **(D)** shoot Si.

The contents of Cu^2+^ in each tissue of wheat seedlings are represented in **Figure 8**. The results showed that Cu^2+^ application increased Cu^2+^ concentration in roots and shoots and the highest concentrations were found in the treatment of Cu^2+^ (**Figures 8A, B**). However, the Cu^2+^ contents in leaves were much lower in SiNP+Cu^2+^ than that in Cu^2+^ treatment alone. SiNP+Cu^2+^ treatment decreased Cu^2+^ in leaves by 26.29% as compared to Cu^2+^treatment alone. SiNP+Cu^2+^ treatment decreased the TF (transfer factor) from 0.56 to 0.31. Moreover, SiNPs addition increased the Si concentrations in both roots and shoots, however, higher concentrations were found in the roots (**Figures 8C, D**).

## Discussion

Heavy metal pollution, particularly Cu^2+^, adversely affects plant growth and productivity (Keller et al., 2015; Huang et al., 2021). The results of our study showed that Cu^2+^ stress resulted in a significant decrease in the chlorophyll contents as well as inhibition of root growth compared with the control group. It has been reported that photosynthesis is impaired due to the accumulation of Cu^2+^ ions and the production of reactive oxygen species (ROS) (Zeng et al., 2021). These reactive oxygen species disrupt the structure of chloroplasts and reduce the activity of key photosynthetic enzymes. Cu^2+^ toxicity in our study greatly reduced photosynthetic traits including Pn, Tr, and gs. Similar findings were reported by Ashraf and Harris (2013), who observed that photosynthesis was inhibited due to damage caused by heavy metals to photosynthetic pigments. Consistent with their findings, our study found that chlorophyll a and chlorophyll b contents in wheat leaves were significantly reduced under Cu^2+^ stress. It has been well established that excessive ROS production has been shown to lead to chlorophyll degradation (Tamaka and Tanaka, 2006; Malik et al., 2022). Heavy metal may cause deficiency of essential nutrients and Fe and Mg deficiencies resulting from heavy metal stress (necessary for chlorophyll synthesis) may affect the synthesis of chloroplast in leaves, which may also lead to a decrease in pigment concentration (Seregin and Kozhevnikova, 2006; Zeng et al., 2021). Our results are consistent with the findings of Vieira Filho and Monteiro (2020) which indicated that Si addition increased chlorophyll content under Cu^2+^ toxicity in *Panicum maximum* cv. Tanzania. A previous study showed that Si has a significant role in the alleviation of abiotic stress in plants (Zhang et al., 2021). In our study, as a result of exogenous SiNPs, wheat seedlings were able to grow more rapidly and had increased chlorophyll contents. Plants under Cu^2+^ stress showed a significant decrease in root and shoot FW, plant height, and root length (**Figure 1**). However, SiNP+Cu^2+^ treatment increased the FW of roots and shoots as well as shoot height and root length of wheat seedlings (**Figure 1**). The reason for this increase in plant biomass might be due to decreased uptake and translocation of Cu^2+^ to shoot of wheat plants. Furthermore, decreased concentration of Cu^2+^ in wheat tissues also indicates an alleviating effect of SiNPs on Cu^2+^ toxicity.

It has been shown that heavy metals such as Cu^2+^ can interfere with the normal metabolism of plants, which can lead to the accumulation of ROS such as ${\text{O}}_{2}^{•-}$ and H_2_O_2_ (Gill and Tuteja, 2010). High accumulation of ROS can cause lipid peroxidation (MDA) and degradation of biological macromolecules as well as induce a large amount of unsaturated fatty acid production, which eventually leads to cellular oxidative damage (Berni et al., 2019). There have been several mechanisms developed by plants for scavenging ROS accumulation, including antioxidant defense systems such as SOD, POD, and CAT which can maintain cellular homeostasis (Asada, 1992; Li et al., 2011; Antoniou et al., 2017). In addition, it has been shown that abiotic stress can be alleviated by enzymatic and non-enzymatic antioxidants involved in the AsA-GSH cycle (Ahmad et al., 2018). The results of our study demonstrated that Cu^2+^ toxicity increased membrane lipid peroxidation (MDA) and oxidative stress. Similar results were also reported by Zeng et al. (2021) in wheat under Cu^2+^ toxicity. In our current study, SiNPs treatment significantly reduced the production of ROS in wheat seedlings which are indicated by decreased MDA and H_2_O_2_ contents. The reason for this decrease in MDA and H_2_O_2_ contents could be related to the improved antioxidant system which maintained a balance between the generation of ROS and the scavenger of free radicals in the plant during growth and development under heavy metal stress (Singh et al., 2016; Morkunas et al., 2018; Berni et al., 2019). Our results are in line with the study of Kim et al. (2014) who reported that Si improved the antioxidant defense system and reduced membrane peroxidation in rice plants under heavy metal stress.

The activity of SOD is considered one of the most important first-line defenses in plants by dismutating superoxide radicals into hydrogen peroxide (Saleem et al., 2020; Kamran et al., 2021). SiNPs increased the activity of SOD under Cu^2+^ toxicity. A previous study also reported similar results that Si application under Cu^2+^ toxicity in *Arabidopsis* and cotton (Khandekar and Leisner, 2011; Ali et al., 2016). The same trend was also evident in our results with regard to the increase in SOD and CAT activity which could break down H_2_O_2_ into water and oxygen (Das and Roychoudhury, 2014). Our results showed that SiNP+Cu^2+^ increased the activities of CAT, POD, and reduced contents of H_2_O_2_ indicating actively converting H_2_O_2_ to H_2_O, hence reducing the oxidative stress caused by Cu^2+^. In addition, Pirooz et al. (2021) exposed salvia to Cu^2+^ and found that 1 mmol L^- 1^ Si application had a positive response in activating CAT and SOD activities. Our results are consistent with the study of Vieira-Filho and Monteiro (2022) that Cu^2+^ toxicity decreased the antioxidant defense system while the application of Si increased the antioxidant defense system in Tanzania Guinea (*Panicum maximum* cv. Tanzania) grass. Gilabel et al. (2014) exposed Tanzania guinea grass plants to Cu^2+^ up to 1000 μmol L^−1^ which resulted in a drastic decrease in biomass production due to oxidative stress, identified by the increase in the contents of MDA and H_2_O_2_. Similar results were reported in rice plants exposed to 100 μmol L^−1^ Cu^2+^ (Kim et al., 2014).

It is crucial to maintain an adequate pool of ascorbate (AsA)-glutathione (GSH) under stress conditions (Nahar et al., 2016). AsA is a non-enzymatic and water-soluble antioxidant that interacts directly with ROS in a cell and can reduce the accumulation of ROS in the cell (Al Mahmud et al., 2018). GSH is one of the most important non-antioxidants in the plant body and serves as a stress indicator. In our study, it is interesting to note that a significant increase in SOD, POD, and CAT activities was associated with the increase in AsA-GSH contents under SiNPs (**Figure 6**). The increase in GSH contents might be related to the complexation and detoxification of Cu^2+^ in the vacuoles which are also evident in the decreased concentrations of Cu^2+^ in the upper part of plant tissues. It is interesting to note that concentrations of Cu^2+^ in the roots of the plant in the treatment of SiNP+Cu^2+^ were higher than in the treatment of Cu^2+^ which could be due to the plant tolerance mechanism. Plants retain a high concentration of heavy metals in the roots, therefore, resist their movement and translocation to the upper parts of plants. The non-enzymatic antioxidant glutathione (GSH) produces thiol compounds with relatively low molecular weights (Berni et al., 2019) which not only decrease ROS within the body (Szalai et al., 2009) but are also involved in the formation of phytochelatins (PCs), and complexes with toxic metals in the cytosol. These cytosolic toxins are transported into vacuoles for detoxification, thus reducing the toxicity (Lin et al., 2012; Seth et al., 2012).

## Conclusion

The results of the current study showed that Cu^2+^ stress inhibited root length and shoot height as well as corresponding fresh weights. Moreover, Cu^2+^ stress caused membrane peroxidation (MDA) and high contents of H_2_O_2_ in root tissues which are correlated with high Cu^2+^concentrations in wheat tissues. However, SiNPs application improved plant growth parameters and also reduced the accumulation of MDA and H_2_O_2_ contents by regulating activities of the antioxidant defense system. The improved defense system and reduced Cu^2+^ concentrations might have led to the increasing growth of wheat seedlings under Cu^2+^ stress by SiNPs, suggesting a mitigating effect of SiNPs on Cu^2+^ toxicity. This study provides a valuable reference for the application of exogenous SiNPs to mitigate the Cu^2+^ toxicity in Cu^2+^ contaminated soil. Future research should be focused on the natural environment and molecular mechanisms involved in the SiNP-induced alleviation of Cu^2+^ toxicity should be considered.

## Data availability statement

The original contributions presented in the study are included in the article/supplementary material. Further inquiries can be directed to the corresponding authors.

## Author contributions

MR and SZ designed and supervised this study; MR and MK conducted the experiments; MR, NUR, AP, and MS performed data interpretation; MR, SZ, and MK drafted the manuscript; FA-H, CM, AA-G, ME, and NUR helped in data validation. MR, FM-P, SA, and SZ critically reviewed the final manuscript. FA-H, SA, SZ, ME, and FM-P supervised the research project. SZ, AA-G, CM, and ME got financial support. All authors read and approved the final manuscript.

## Funding

Researchers Supporting Project number (RSP2022R483), King Saud University, Riyadh, Saudi Arabia. This project is financially supported by Science and Technology Program of Guangzhou, China, under the grant number: 202102020229.

## Acknowledgments

The authors extend their appreciation to the Researchers Supporting Project number (RSP2022R483), King Saud University, Riyadh, Saudi Arabia, for providing help to publish this research work.

## Conflict of interest

The authors declare that the research was conducted in the absence of any commercial or financial relationships that could be construed as a potential conflict of interest.

## Publisher’s note

All claims expressed in this article are solely those of the authors and do not necessarily represent those of their affiliated organizations, or those of the publisher, the editors and the reviewers. Any product that may be evaluated in this article, or claim that may be made by its manufacturer, is not guaranteed or endorsed by the publisher.
